# Mutual Role Expectations by Patients and General Practitioners—A Mixed Methods Study on Complementarity

**DOI:** 10.3390/healthcare10102101

**Published:** 2022-10-20

**Authors:** Barbara Plagg, Adolf Engl, Giuliano Piccoliori, Hermann Atz, Ulrich Becker, Johann Kiem, Verena Barbieri, Klaus Eisendle, Christian Josef Wiedermann, Susanne Ursula Elsen, Walter August Lorenz

**Affiliations:** 1 Institute of General Practice and Public Health, College for Health Care Professions—Claudiana, 39100 Bolzano, Italy; 2 Faculty of Education, Free University of Bolzano, 39100 Bolzano, Italy; 3 Apollis—Institute for Social Research and Opinion Polling, 39100 Bolzano, Italy; 4 Department of Public Health, Medical Decision Making and HTA, University of Health Sciences, Medical Informatics and Technology—Tyrol, 6060 Hall in Tyrol, Austria; 5 Department of Applied Social Sciences, Faculty of Humanities, Charles University, 18200 Prague, Czech Republic

**Keywords:** patient’s role, participatory behavior, medical consultation, doctor–patient relationship, health literacy

## Abstract

Background: Changes in public attitudes toward “authorities” in general, as well as shifts in medical practice toward participative models of diagnosis and treatment, imply fundamental transformations in the patient–doctor relationship. However, consistency in reciprocal role expectations cannot be assumed, and this study reveals significant discrepancies in attitudes and behaviors in primary health consultations. Methods: We conducted a study in the tri-lingual northeastern Italian region of South Tyrol to determine whether perceptions of the patient’s role were congruent or differed. In a mixed method approach, the quantitative research part consisted of a survey with 34 identical questions for general practitioners (*n* = 109) and adult primary care patients (*n* = 506) on verbal communication, self-initiative and health literacy, interpersonal and social qualities of the patient–physician relationship, and formal aspects of the consultation. Patients were interviewed via telephone, and general practitioners responded online. In the qualitative part, 26 semi-structured in-depth interviews were conducted with the patients and analyzed. Results: General practitioners considered patients’ communicative efforts (*p* < 0.001), self-initiative (*p* < 0.001), compliance (*p* = 0.0026), and openness regarding psychosocial issues (*p* < 0.001) to be significantly more important, whereas patients showed a tendency to give increased importance to formal aspects such as politeness and hygiene (*p* < 0.001). Perception of the patient’s role differed significantly between the Italian and German linguistic groups. Conclusions: Patients and general practitioners differ in their understanding of patients’ roles. These data suggest that a considerable proportion of the population lacks a clear and tangible idea of the active role they could play in consultations. Targeted information on the identified aspects of patient–provider communication may facilitate participatory behavior and positively impact the longitudinal quality of the patient–general practitioner relationship.

## 1. Introduction

This study took place against the background of changes in public attitudes toward professional authority, calls for greater citizen self-determination, and the proliferation of advice-giving information through digital channels. Together with an increased emphasis on patients’ rights in medical treatment [1], we assumed that these contextual factors have a profound impact on the doctor–patient communication and relationship, particularly at the frontline of medical diagnosis in general practice. By elaborating on a preparatory aspect for the further development of patient-centered approaches to medical care, we aimed at identifying the components of mutual role expectations of patients and GPs so as to identify symmetries and discrepancies. In this way, we hope that our study can help to identify and promote constructive participative role models for patients and doctors in primary care consultations and, hence, identify forms of communication that facilitate participative treatment modes. There is growing literature on the co-production of knowledge, particularly the importance of informal knowledge on the part of patients, which raises the question of how professionals can bring their expertise to bear [2]. Patient-centered care models [3] that acknowledge the important role of agency in patients are displacing disease-centered biomedical models [4] with their associated paternalism [5] and are considered to bring better health outcomes, greater patient satisfaction, and reduced health costs [6,7,8]. This is reflected in the changed modes of patient–provider communication that have moved beyond the traditional practices of information transfer (based on a one-way monologue) toward a notion of information exchange (based on two-way dialogue) considered to bring better results [9].

From a sociological perspective, these developments took place in the context of the widespread and growing movement of service user involvement in social and health services [10] which developed during the 1980s. While the drive behind many of these movements, particularly in the areas of disability and mental health, came initially from self-help or relatives’ initiatives [11], later influences can be traced back to consumerism and the liberalization of economic relations, which also affected public services. In the course of the privatization of many previous state monopolies, the “voice of the consumer” became a factor that increasingly had to be taken into consideration in the planning and management of services, with all the associated ambiguities [12]. On one hand, this increased “user choice” among competing service offers; on the other hand, it promoted a division between better equipped private facilities that could indeed offer user-oriented choices and a residual sector of services catering for users unable to pay for such a wider choice [13]. Therefore, the broader socio-political context of health services must be considered when promoting particular forms of patient involvement.

Furthermore, the shift toward patient participation gives rise to considerations concerning the degree and reliability of self-knowledge that patients can bring to a consultancy under the heading of ‘health literacy’ [14,15] which in turn makes it important to examine the conditions under which relations of trust can be established [16,17,18,19]. The success of reaching consensus over shared health goals and, hence, compliance with suggested treatments depends on the reciprocity of role expectations [20], which in turn are strongly influenced by sociocultural contexts that define accepted norms. Role expectancies vary between countries and among different cultural groups [21,22,23] and contain a strong gender dimension [24].

While there is a considerable body of literature documenting the current patient expectations of their doctors (e.g., [25,26,27]), little is known about the expectations of the role and behavior of patients on the side of general practitioners (GP), so that inconsistencies in expectations and their impact on patient–GP relationships could be identified and practice consequences drawn.

The reality of our study was heightened during the COVID-19 pandemic during which the trustworthiness of medical advice and compliance with health protection measures were challenged by campaigners against compulsory vaccination [28] and the proliferation of conspiracy theories on social media. These reactions cast serious doubts on the extent to which health literacy has increased through access to digital resources. These developments make it imperative to regard advances in health literacy not just as a matter of ‘better public health education’ but also as an invitation to critically examine the role of digital and social media that can contribute to or undermine trusting relationships in medical consultations and indeed challenge the entire notion of medical expertise [29]. It seems that the quality and reliability of ‘medical knowledge’ on the side of professionals and on that of patients can no longer be determined with reference merely to ‘objectivity’ but also needs to take into account the embeddedness of such knowledge in social values and hence of the influence of powerful societal and political interests [30].

Our study therefore started from the basic assumption that GP consultations serve the purpose of co-constructing person- and situation-relevant medical knowledge and thereby constitute important moments of enhancing ‘critical health literacy’ on both sides. Our approach reflects Nutbeam’s [15] proposal to integrate cognitive, interpersonal, and social skills in medical consultations. Crucial in this agenda is the promotion not only of e-literacy generally [31] but also of appropriate modes of communication between patients and GPs [32] and the capacity building of patients to take an active role in shared medical decision making, which has been slow and difficult [33]. Patients can only have more control over their health, as the Ottawa Charter for Health Promotion [34] proposes, if they are equipped with the means to identify personally relevant, meaningful, and achievable health objectives and the corresponding resources to realize them. GPs play a critical role in steering the course between treating patient participation merely as a response to consumer demands and retreating to a paternalist insistence on scientific evidence [35].

Therefore, we hypothesized that discrepancies in role expectations between patients and GPs would adversely affect communication processes and, hence, the establishment of trust [19] and the degree of compliance. We aimed to identify the main areas in which discrepancies in role expectations arise and explain their causes.

Our survey was based on Mead and Bower’s [36] five key dimensions of the patient-centered approach: (a) having a biopsychosocial perspective of the patient, (b) understanding the patient as a person, (c) sharing power and responsibility within the physician–patient relationship, (d) building a therapeutic alliance between the physician and patient, and (e) understanding the doctor as a person. We wanted to shed light on the components of expectations that define the respective roles, functions, and responsibilities of patients and GP as seen from both sides, as well as respective preferences for factors that can facilitate understanding during consultations in terms of communication styles, presentation of information, and structural contexts. We consider these aspects, which were detailed in the questionnaires and interview guidelines, as crucial preconditions for better mutual understanding, relationship building, and the formation of a therapeutic alliance. GPs cannot anticipate that all patients have or wish to have greater involvement in and control over treatment forms as their priority, as evidenced in the study by Say, Murtagh, and Thompson [37]. These differences may not simply reflect personal preferences but are influenced by cultural factors. In this regard, we took advantage of the particular geopolitical situation of our study territory of South Tyrol in northeastern Italy, which is characterized by legally sanctioned equal recognition of the region’s three languages, German, Italian, and Ladin. Additionally, we aimed to understand whether sociocultural factors such as gender, education, and work experience influence the understanding of the patient’s role within medical consultation.

### Research Objectives

The present study aims to:

1. Identify the main areas in which discrepancies with regard to the patient’s role in primary care consultations arise by comparing patients’ and GPs’ expectations of the patient’s role;
2. Analyze whether sociocultural factors such as gender, education, and work experience modulate the narrative of the patient’s role within medical consultation.

## 2. Methods

### 2.1. Study Design

Because of the complexity of the investigated social phenomena and interactions, we combined qualitative and quantitative approaches in the present study to utilize their complementary and mutually corrective results. More specifically, we applied an exploratory sequential design in which collection and analysis of quantitative data proceeds collection and analysis of qualitative data. This enabled us to obtain significant issues and indicators in preparation for a deeper understanding of their significance through subsequent qualitative interviews which in turn we correlated critically with the quantitative data. The present mixed methods study was therefore conducted in two phases: the first phase was a quantitative survey conducted on 506 patients and 109 GPs. In the second phase, 26 patients participated in individual in-depth interviews to elaborate on findings generated in the population-level survey. Going then back to the quantitative data allowed us to attribute more general significance to the personal statements of informants, for instance with regard to the impact of linguistic, cultural or educational background. This design was chosen since starting with the quantitative strand, followed by textual data, allows one to use narrative data to explain and interpret numeric findings and vice versa, providing thus a comprehensive understanding of the investigated health-related patterns of role expectations and communication forms [38]. Consensus-based checklist recommendations for the reporting of survey studies (CROSS) were followed [39].

### 2.2. Quantitative Part

#### 2.2.1. Sample

Patients: Data were obtained from a population-based cross-sectional telephone survey study. The eligibility criteria for participants were living in South Tyrol, possessing a private landline, being at least 18 years old, declared a member of either the German or Italian linguistic group, and having seen a family physician at least once during the last 12 months. Computer-assisted telephone interviews were conducted between 13 and 26 April 2018. Data collection was conducted by Apollis (www.apollis.it), a private research institution in Bolzano (BZ), Italy, which conducts empirical studies focusing on education, labor market topics, and active aging. Professional interviewers contacted a random sample of 1272 households in their preferred language. After excluding phone numbers that were wrong or not reachable, the remaining persons were invited to participate in the telephone survey if a disease-related visit to their GP within the last 12 months had taken place and appointments were made to answer the phone survey. Ultimately, 506 valid interviews were conducted with adults (≥18 years). The response rates of households and eligible patients were 31% and 57%, respectively.

An iterative weighting adjustment to correct for non-responses was performed for sex, age, and geographic region according to ISTAT data [40]. The survey was conducted using a questionnaire with a maximum duration of 20 min.

General practitioners: We contacted 311 registered GPs via mail with 109 GPs that eventually filled out the online survey. Although we aimed for extensive participation, the response rate was only approximately 35%. Because factors such as work experience and specialization are considered more important than geographical area and sex, no weighting adjustment was made. The representativeness of the GP sample cannot be guaranteed.

#### 2.2.2. Questionnaire

The final questionnaire for the quantitative part included items developed within a focus group with professionals (consisting of Italian and German-speaking GPs (eight participants) and social scientists (three participants)), items taken from the “Patient Consultation Values questionnaire” (PCVq) and of a questionnaire of a multicenter European study, GULiVER [27]. Overall, we identified four overarching thematic clusters as key elements of the patient–doctor relationship [41]: (i) verbal communication, (ii) self-initiative, health literacy, and preventive measures, (iii) interpersonal and social qualities of the relationship, and (iv) formal aspects of the visit. The final form for the quantitative part consisted in a set of 34 identical questions for GPs and patients with respectively adapted change in perspective (i.e., for the patient: “How important do you find asking your GP for clarification in case you did not understand well?” For the GP: “How important do you find it that your patient asks for clarification in case he/she did not understand well?”). The answers were Likert-scale with four points to depict a clear opinion tendency, except for one question regarding self-initiative (five points). Two open-ended questions were asked to complete the survey.

#### 2.2.3. Statistical Analysis

To provide an overview of the most divergent and concordant items, we focus the analysis of the present paper in most cases on the best (“most important“) and worst (“least important“) rated items. Since data are ordinal level scaled, results are expressed as percentages, and independent groups were compared using 95% confidence intervals for differences in proportions (between percentages) and chi-square tests. For the variables “educational level” (5 different items) and “work experience” (six items), we allowed analysis for all items. Thus, in these cases, post hoc testing using chi-square tests was necessary to obtain information about the differences between single items. For post hoc tests, *p*-values were adjusted for multiple testing. To facilitate the interpretation of the results and account for missing values, we present the absolute number of unweighted cases for all results in the tables. All statistical analyses were performed using R version 3.6.2.

### 2.3. Qualitative Part

#### 2.3.1. Sample and Analysis

Patients were recruited at the GP’s offices by two of the authors (JF and SR), who informed the respective GPs about the survey and provided the participants with information about the study. Following informed consent, data collection was performed through individual semi-structured face-to-face interviews with 26 patients (19 German-speaking participants and 7 Italian-speaking participants; none of the participants were known to the researchers before). The interviews were audio recorded and transcribed. Data were analyzed using qualitative content analysis [42]. Accordingly, the interviews were coded, grouped into categories, and analyzed. Each transcript was coded by two researchers to ensure reliability, while further interpretation and discussion took place within the entire research team to ensure that the analytical deductions were congruent with the extracts.

#### 2.3.2. Questionnaire

For the semi-structured interviews, the research group developed a patient interview guide with open-ended questions based on the four macro areas identified within the focus group. The questionnaire was translated into German and Italian. Demographic data were collected prior to the interviews. Further personal data of the interviewees resulting from the interviews were anonymized during the course of the transcription.

## 3. Results

### 3.1. Quantitative Part

#### 3.1.1. Composition of the Two Samples (Patients and General Practitioners)

The patient cohort (*n* = 506) was representative of all patients in South Tyrol with respect to the variables controlled by the weighting procedure: age, sex, and geographic distribution. More German-speaking and female physicians participated in the GP cohort survey (*n* = 109) (Table 1).

#### 3.1.2. Concordance and Divergence between Patients and Their General Practitioners

The majority of patients (79%) believed that their GPs’ answers to the items raised in the survey coincided largely with their own answers, while only half of all GPs (48%) thought so (95% CI for difference in proportions [0.21;0.42]; *p* < 0.001). The picture that emerged generally is that both protagonists perceive the patients’ roles differently and that their expectations do not coincide in several aspects (Table 2). GPs put more weight on particular communicative aspects, self-initiative, healthy lifestyle, and openness toward considering psychosocial issues (Table 2). On the other hand, patients tended to attribute more importance to formal aspects of medical consultation, such as politeness and cleanliness (Table 2). These incongruences were differently pronounced between the German and Italian groups of patients and GPs (Table 2). Language group, education level, work experience, and sex further determined these variations (Table 2, Table 3 and Table 4), whereas geographical location (rural vs. urban) did not significantly influence the perception of the patient’s role within our cohort (Table 3).

#### 3.1.3. Interpersonal Qualities of the Relationship: Trust and Honesty

In the qualitative interviews, both patients (40%) and GPs (51%) pointed out that trust is the most important aspect of doctor–patient relationships. Honesty and openness ranked second among the patients (22%) and GPs (38%), respectively. Quantitatively, both GPs and patients confirmed the importance of honesty, with Italian-speaking patients and GPs giving honesty significantly more importance than did German-speaking patients (95% CI [2.1;17.1]; *p* = 0.012) and GPs (95% CI [8.3; 41.2]; *p* = 0.004, Table 2).

However, while patients consider honesty important, on closer inspection, they are not willing to be honest in every respect; they weigh the importance of talking about psychosocial issues (95% CI [17.8; 33.9]; *p* < 0.001), giving information about their own research (95% CI [6.1; 21.1]; *p* < 0.001), own presumptions (95% CI [6.8; 21.8]; *p* < 0.001), self-medication (95% CI [21.2; 36.5]; *p* < 0.001), negative feedback (95% CI [9.1; 24.4]; *p* < 0.001), and results of other medical consultations (95% CI [8.8; 24.1]; *p* < 0.001, Table 2) significantly lower than their GPs.

Within subgroups, we found more readiness to talk about psychosocial issues among the higher-educated groups than the lower-educated groups (*p* < 0.001, Table 4). On the professional side, we found that female GPs rated the importance of openness regarding psychosocial problems significantly higher than their male colleagues did (95% CI [9.4; 43.6]; *p* = 0.002, Table 3). Interestingly, with increasing work experience, GPs considered honesty (*p* < 0.001; Table 5), information about self-medication (*p* = 0.0023), and other consultations to be significantly less important than their younger colleagues (*p* = 0.003).

#### 3.1.4. Verbal Communication

GPs (Table 2) considered that patients should come forward with questions for clarification to be significantly more important than the patients themselves (95% CI [20.1; 4.1]; *p* < 0.001). We observed gender differences on the professional side; female GPs found it significantly more important that patients ask for clarification (Table 3; 95% CI [6.1; 34.7]; *p* < 0.0011) than their male colleagues. When examining our two cultural subgroups, the Italian-speaking patient cohort showed increased readiness to demand clarification (95% CI [6.6; 22.5]; *p* < 0.001) compared to the German-speaking group.

#### 3.1.5. Self-Initiative, Health Literacy and Preventive Measures

We found a statistically significant discrepancy between doctors’ and patients’ perceptions of self-initiative in preventing diseases and maintaining health (95% CI [13.2; 29.4]; *p* < 0.001). GPs consider lifestyle to have a very important impact on a patient’s health, whereas patients consider their influence on their own health status to be significantly lower. Interestingly, we observed a social gradient in relation to health literacy on the patient’s side. Within the group with the lowest education level, only 28% attributed importance to their own contributions to maintaining or improving their health status. Remarkably, the conviction that self-initiative is important increased with each educational level (*p* < 0.001, Table 4). We also observed that GPs found it significantly more important that patients knew their current medications (Table 2; 95% CI [13.6; 28.4]; *p* < 0.001) than the patients themselves.

Information gathering outside of the medical consultation was rated differently by the two linguistic groups. Italian-speaking patients and GPs displayed a significantly negative attitude toward self-information compared to German-speaking patients (95% CI [30.4; 3.6]; *p* < 0.001) and GPs (95% CI [42; 73.2]; *p* < 0.001). In addition, 53% of all Italian-speaking GPs, compared to 7% of all German-speaking GPs, did not want their patients to have access to independent and trustworthy information (95% CI [30.5; 61.7]; *p* < 0.001). The importance of compliance was rated significantly higher in the patient cohort with the highest education level than in that with a lower educational background (*p* < 0.001, Table 4).

#### 3.1.6. Formal Aspects of the Medical Consultation

Strikingly, 72% of all patients thought that personal hygiene was very important, whereas only 24% of GPs concurred with this valuation (95% CI [−54.9; −40.3]; *p* < 0.001). Most patients (62%) find “being friendly” very important compared to only 36% of all GPs (95% CI [−34.7; −18.6]; *p* < 0.001). Overall, GPs give more importance to aspects that they consider to have an immediate and tangible impact, such as giving information regarding self-medication or their own presumptions, while patients consider relational aspects to be rather important.

### 3.2. Qualitative Part

#### Patients on Their Role within Primary Care Setting

Within the quantitative survey, it became evident that patients and GPs diverge in their opinions, as they assign importance to divergent aspects with regard to the patient’s role. Subsequently, the qualitative strand helped to identify reasons for the observed discrepancies and aimed to further understand whether and what kind of active part patients see for themselves before, during and after consultation.

Prior to consultations with their GPs, half of the interviewees indicated that they had performed an online health search. Additionally, many first consulted specialists within the private healthcare sector, first aid departments or resources, pharmacists, or people with medical expertise within their circle of relatives. A smaller proportion of all interviewees stated that they did not prepare for a visit; however, within the course of the interview, it became apparent that they nonetheless thought about how they would describe symptoms in advance and made efforts to define symptoms as accurately as possible in front of their GP. Overall, the qualitative interviews show that most respondents think in advance about the questions and concerns they want to present to their GP; however, no one carries out this preparation in written form. However, younger patients proposed taking notes as a strategy for older patients.

“If you have a major complaint (…) key points would help you” (K2, 31–32); “In my opinion, the GPs don’t have much time, so the patients might forget something. If written down, they are more likely to answer” (K7, 43–45); “Maybe someone goes to the doctor and forgets their questions. Then it would be helpful just to write them down” (K11, 55–56).

Communication during the visit was generally described as comprehensible, although the information provided by the doctor was sometimes perceived as too superficial and lacking in empathy. According to several patients, this was mainly due to time pressure and work overload resulting from the large number of patients.

“Often, GPs are overburdened and have insufficient time to respond to patients. Patients often do not ask many questions either.” (K2, 38–39); “When there are a lot of people waiting outside and you have the feeling that it is not so important what I say…” (K6, 68–69); “There is always the pressure that someone is waiting outside. It is also stressful for the patient when he knows that there are still 20 people waiting outside, and he would still have three questions.” (R12, 36–38); “Mostly there is not enough time, so you have to see that you get through quickly” (R7, 36–37).

Overall, patients are convinced that the success of communication depends on the efforts of both the GP and the patient, and that they seek to play an active part during consultation. However, beyond providing a verbal description of symptoms and concerns, patients react surprised when asked how they could provide further concerns and clarifications without being asked by the GP.

“I must honestly say that I don’t really concern myself with that.” (K8, 49); “I think that the doctor should be the active part.” (K10, 52–53); “I do not think that is really my job. It is the GPs’ job of engaging with the patients. Now it’s not the patients who will have to be taking the initiative that’s totally the wrong way for me” (R1, 59–61).

We noticed time and again that patients responded to the question of what other contributions they themselves could make to the consultation by diverting and giving instead further details of what they wished the GP would further do.

“The GP should have an overview, to know the patient better, about everything, even the patient’s background“ (K9, 45–55).

Some patients clearly stated that they had not thought about what contribution they could make.

“I can’t say anything about that“ (K3, 51); “I can’t say. I sometimes see other patients who leave unsatisfied, but in my opinion, it is the patient’s problem“ (R10, 42–43).

The observed discrepancies in the quantitative strand can be linked to and explained in parts with a general vacuum in the understanding of the patient’s role. While most patients recognize that the success of the visit depends on what both parties involved contribute, the qualitative survey makes clear that they have no concrete suggestions for how to play their role apart from asking questions during the visit and making efforts to adhere to the recommended therapy afterwards. Overall, their understanding of their active roles remains incoherent and they seem to have not been equipped with a repertoire of relevant and achievable roles before, during and after medical consultation that would facilitate their greater active participation.

“Yes, I think that the patient can do something: By not just going there and staying superficial“ (K7 58–59). “Yes, the patients could actually contribute to optimization” (K2 76).

Ultimately, when asked about suggestions that would help improve the consultation, the proposals made by all interviewed patients exclusively related to the doctor’s side, while none of the 26 respondents made suggestions for the patient’s side.

## 4. Discussion

Patient trust in GPs is fundamental to effective clinical encounters [16]. We found that patients presume honesty and trust to be a minimal condition for a successful patient–doctor interaction, even against the background of changed societal norms due to the increased availability of information and other recent societal phenomena, such as greater self-confidence and emphasis on autonomous decision making. Associations between patients’ trust and their perceptions of communication within the consultation have been identified and facilitated by a range of organizational and personal factors [18,43]. However, this presupposes symmetrical or complementary role expectations on both sides, which, as our study found, are not necessarily given in general practical situations. In view of wider societal changes toward greater self-assertion, our patients tend to still conform to a well-defined narrative of a “good patient,” and in this regard, their understanding of the patient role differs from that of their GPs. In line with other surveys [26,44], the patient’s role has not yet assumed new and definite contours. This can be broken down into discrepancies between the patients’ and GPs’ views within all four domains that define the patients’ roles, i.e., verbal communication, formal aspects, interpersonal qualities, and health literacy. In specific consultation situations, patients are likely to adopt a rather avoidant and defensive attitude and seem unwilling to proactively present information that could be relevant for a wider understanding of their situation. This concerns information of a formal, anamnestic, or psychosocial nature and hence indicates a limited willingness before, during, and after the visit to render the encounter collaborative and participative. These attitudes fall short of their GPs’ expectations in many domains and hence constitute impediments to the full development of the collaborative potential in medical consultations enshrined in new models of general medical practice. Our qualitative findings suggest that the reason for this may be found in the fact that apart from accurate symptom descriptions and questions in cases of comprehensive difficulties, patients have little concrete idea of what active part they can play in the consultation, so that health literacy and self-assertiveness cannot simply be supposed to be present in the general population. Nevertheless, we found that among patients who had better educational opportunities, there was more self-confidence in engaging more actively in exchanges. GPs seem to compensate for these differences in readiness to participate, since we found that older participants tended to attribute less significance to honesty (Table 5), suggesting that with more extensive clinical experience, they can infer more details concerning a patient’s personality traits, family situation, and lifestyle. Therefore, the conditions under which active participation in diagnosis and treatment can be strengthened warrant further examination.

When patients withhold information from doctors, this can clearly incumber medical diagnosis and treatment [45]. This is particularly relevant in primary care settings, which form a crucial stage of decision making over future treatment pathways. Attention needs to be given to the concerns that primary care patients might have that affect and sometimes limit their disclosure [46]. For instance, patients tend to value formal aspects, such as politeness and physical cleanliness, and put more weight on socially expected and positively connoted behaviors. Patients withhold information (e.g., on self-medication, own presumptions) that they consider unimportant and/or which may, according to their judgement, negatively affect their GPs’ opinions and attitudes toward them [47]. In contrast, GPs find obtaining information regarding psychosocial issues or self-medication highly relevant, which is information that for patients might be potentially sensitive or discriminating. When patients misjudge the importance of sharing information, that is, on self-medication or their own presumptions and attempts at treatment, this points toward perceived and real discrepancies in power and the presence of discriminatory practices prevalent in society that might not be expressed by the GP but are being projected onto the relationship on the basis of general life experiences. Contextual issues such as gender, socialization, and work experience of GPs further determine the degree to which patient and GP expectations concur. Educational level on the part of patients, for instance, correlates to the attitude toward the impact of lifestyle on health status, with higher educational level associated with a more positive view on lifestyle and self-efficacy. Likewise, readiness to ask for clarification, talk about psychosocial issues, and be compliant increases with each higher level of education.

It is well known that patients tend to rarely verbalize their emotions directly but instead offer clues that they only elaborate if invited [48]. Our data show that patients often seem unaware of the importance of psychosocial information and might be too ashamed to share this with their GPs or be hindered by structural barriers, such as time pressure. GPs can engender a substantial increase in such disclosure by adding one or two questions about mood or interpersonal problems in their clinical interviews [49] and, as our data suggest, by relating the importance of such information sharing to the creation of a trusting relationship and by showing an understanding of the fears surrounding such disclosures.

The different factors influencing a patient’s behavior are heterogeneous and difficult to individualize. Behavioral differences may also be due to individual sociocultural perceptions of patients’ roles [50]. Within our cohort, patient–GP discordance was differently pronounced among people with diverse cultural backgrounds. Ethnic groups, of course, are not homogenous entities, and language only determines generalities and cultural universals. However, our study underlines how susceptible patient–doctor interactions are to the respective sociocultural backgrounds of both protagonists.

We are aware of the limitations of this study arising from the specific characteristics of this sample undertaken in the relatively small but highly heterogeneous population of South Tyrol on account of its multi-lingual and multi-cultural composition. The limitations of the study extend furthermore to the fact that Likert-scaled items may be liable to bias from confounding factors such as age, education and language but nevertheless represent generally a short and reliable method. Despite ongoing methodological discussions on whether a 4- or 5-point scale is better, we decided on a “forced” scale of 4 points in order to obtain a clear tendency without a “neutral” answer option and to increase the feasibility of the telephone interviews. Being aware of the further limitations arising from the use of a mixed methods approach, we felt it necessary to add a qualitative survey in view for instance of differences showing up between the German and the Italian language groups in the statistical evaluation of our quantitative data which required a more direct engagement with the meanings attributed to certain terms and concepts in the respective language groups. We recognize that this might limit the generalizability of our findings and their application to other language populations but trust that our results can be taken as indicators for further research in specific cultural contexts which inevitably influence the patient–doctor communication and relationship. Further limitations were that we included only 26 Italian GPs compared with 76 German-speaking GPs and that against the background of a high proportion of men working as GPs in South Tyrol, more female GPs participated in this study. Therefore, representativeness was not given for the GP cohort, and because of the lower number of Italian GPs, comparisons also in this regard between the two language groups must be considered with caution.

## 5. Conclusions

Our findings confirm that despite considerable changes in public attitudes toward “authorities” including medical experts and despite widespread departures from paternalist attitudes toward patients in medical practice generally, expectations and role perceptions for patients in primary care consultation still differ in several important respects between the two parties involved, and both sides have different expectations concerning the preparation and presentation of relevant information. Patients continue to expect GPs to take active and leading parts of the interaction, even though a greater degree of participation would be welcome from the medical side. Apart from describing symptoms, asking limited questions, and complying with prescribed therapy, patients are uncertain about how they could engage more in the interchange, thereby improving the quality of the consultation.

These largely unrecognized discrepancies may lead to continued misconceptions on both parts, and hence, to non-compliance in therapy, unnecessary investigations, and second opinions. They also pointed out that the overall contribution of patients as a resource cannot be utilized efficiently under these circumstances [51] and that the further development of patient-centered medical practice needs to pay close attention to these discrepancies. Increases in health literacy appear to be unevenly distributed in society and can only become effective once they are promoted, which is in consideration of wider social inequalities in educational levels, public participation, and ethnic and gender identity recognition. Apart from targeted public campaigns, which were launched in the Autonomous Province of Bozen/Bolzano as a result of these findings, stressing the central “medical-curative” role of the patient (in relation to the mere “assisting” role of the doctor, after the famous motto by Paracelsus), the interactions in primary consultations themselves offer opportunities of addressing expectations and thereby transforming existing discrepancies toward “critical health literacy” on both sides.

Knowledge of how to actively participate in a consultation as a patient can be considered a central health competence and opportunity for empowering patients to bring that knowledge to bear needs to be widened beyond the immediate primary health setting to reach educational institutions and the media. Our study sought to highlight the complexity of factors and processes that shape this interaction and to show the practical relevance of drawing scientific attention to the details of these elements as the basis for further studies. Eventually, how well patients and doctors match regarding their respective concepts of the patient’s role in consultation impacts the interactive and longitudinal quality of their relationship and hence the effectiveness of treatment.

## Figures and Tables

**Table 1:** Characteristics of the study sample.

Variable		Percentage
Patients (*n* = 506)		
Educational level	None/primary school	9
	Secondary school	20
	Vocational school	28
	Grammar school	26
	University	17
Language group	German	65
	Italian	30
	Other	5
Gender	Male	53
	Female	47
Occupational status	Employed	49
	Unemployed	3
	Retired	27
	Student	6
	Housewife/househusband	5
	Entrepreneur	9
Composition of households	Single	12
	2 people	31
	3 people	17
	4 people	22
	5+ people	17
Duration of relationship with General Practitioner	<5 years	29
	5–9 years	15
	10–19 years	19
	≥20 years	37
**General Practitioner (*n* = 109)**		
Language group	German	69
	Italian	31
Gender	Female	50
	Male	50

**Table 2:** Comparison between patients and general practitioners, and their respective language groups cited.

Variable		Patients (*n* = 506)				General Practitioners (*n* = 109)				Differences between General Practitioners and Patients ALL *p*-Value ^§^ [95% CI in %]
		ALL	GER	ITA	Differences between Language Groups	ALL	GER	ITA	Differences between Language Groups	
		% (±) ^†^ *n*	%	%	*p*-Value χ^2^ Test ^‡^ [95% CI in %]	% (±) ^†^ *n*	%	%	*p*-Value χ^2^ Test ^‡^ [95% CI in %]	
Verbal communication	Asking for clarification	55 (+) 504	50	65	<0.001 * [6.6; 22.5]	82 (+) 108	83	87	0.7216 [−10.1; 17.7]	**<0.001** [20.1; 4.1]
	Expectations for the visit	21 (+) 499	19	27	**0.0258** [00.9; 14.6]	26 (+) 108	20	40	**0.021** [2.9; 37.1]	0.1563 [−2; 12.3]
	Negative feedback	19 (+) 486	21	17	0.2896 [−10.2; 2.8]	36 (+) 109	39	33	0.662 [−0.233; 0.128]	**<0.001** [9.1; 24.4]
	Results of other consultations	46 (+) 501	41	57	**<0.001** [8.1; 24.2]	78 (+) 108	81	80	1 [−0.165; 0.136]	**<0.001** [8.8; 24.1]
	Own research	21 (−) 494	7	48	**<0.001** [35; 48.2]	10 (−) 108	1	30	**<0.001** [15.1; 42]	**<0.001** [−16.3; −4.9]
	Own presumptions	19 (+) 494	20	18	0.7138 [−8; 5]	33 (+) 108	39	27	0.2108 [−29.4; 5.6]	**<0.001** [6.8; 21.8]
	Self-medication	45 (+) 494	39	53	**0.0114** [60.5; 47.2]	73 (+) 108	75	80	0.6047 [−10.5; 21.2]	**<0.001** [21.2; 36.5]
Health literacy	Lifestyle and personal behavior	38 (+) 504	42	33	**0.028** [−16.7;1]	62 (+) 108	59	70	0.2424 [−6.5; 29.3]	**<0.001** [16.1; 32.2]
	Asking what to do themselves to improve health	30 (+) 493	26	30	0.35 [−0.111; 0.037]	51 (+) 108	50	53	0.8391 [−0.154; 0.221]	**<0.001** [13.2; 29.4]
	Access to trustworthy information	23 (−) 487	13	42	**<0.001** [21.6; 35.7]	21 (−) 107	7	53	**<0.001** [30.5; 61.7]	0.4626 [−9.8; 4.1]
	Self-information through friends, books, internet	20 (−) 500	7	44	**<0.001** [30.4; 3.6]	33 (−) 108	16	73	**<0.001** [42; 73.2]	**<0.001** [6.1; 21.1]
	Know current medicine	56 (+) 501	56	56	1 [−7.8; 7.9]	77 (+) 108	83	70	0.1271 [−29; 3.3]	**<0.001** [13.6; 28.4]
	Compliance	58 (+) 505	56	62	0.0956 [−1.1; 4.8]	70 (+) 109	61	87	**0.0017** [10.3; 41.9]	**0.0026** [4.4; 20]
Interpersonal qualities	Honesty without shame	68 (+) 505	64	74	**0.0116** [2.1; 17.1]	66 (+) 108	59	83	**0.004** [8.3; 41.2]	0.6949 [−9.7; 6]
	Psychosocial issues	30 (+) 498	26	39	**<0.001** [5.7; 20.8]	56 (+) 109	56	63	0.526 [−0.113; 0.253]	**<0.001** [17.8; 33.9]
Formal aspects of the visit	Hygiene	72 (+) 501	68	78	**0.0059** [2.9; 17.4]	24 (+) 107	20	34	0.0998 [−0.025; 0.315]	**<0.001** [−54.9; −40.3]
	Friendliness	62 (+) 501	60	65	0.1817 [−2.4; 3.4]	36 (+) 106	36	34	0.9848 [−0.200; 0.165]	**<0.001** [−34.7; −18.6]

^†^ (+) “Very important,” (−) “Not at all important”; * Significance, *p* ≤ 0.05; ^‡^ H_0_, no dependency between language and item; ^§^ H_0_, no dependency between patient/general practitioner and item. Abbreviations: CI, confidence interval; GER, German; ITA, Italian.

**Table 3:** Gender-specific and urban-rural differences among general practitioners.

General Practitioners (*n* = 109)				*p*-Value [95% CI in %] *
Gender		**Female *n* = 55** **N (%)**	**Male *n* = 54** **N (%)**	
	Asking for clarification (+)	50 (93)	39 (71)	0.0011 [6.1; 34.7]
	Inform about psychosocial issues (+)	38 (69)	23 (43)	0.002 [9.4; 43.6]
	Inform about self-medication (+)	47 (85)	33 (61)	<0.001 [8.4; 40.3]
Residence		**Urban *n* = 44 ** **N (%)**	**Rural *n* = 65 ** **N (%)**	
	Tell own presumptions (+)	11 (26)	25 (38)	<0.001 [8.4; 40.3]
	Friendliness (+)	20 (47)	18 (29)	0.0562 [−36.4; 0.5]
	Give negative feedback (+)	11 (26)	28 (43)	0.0627 [0.4; 34.6]

(+), “very important”. Abbreviations: CI, confidence interval. * 95%—confidence interval for the difference in proportions.

**Table 4:** Influence of patient’s education level on their perception of the patient’s role.

Patients (*n* = 506)							
Educational Level	Primary School (Gr. 1) *n* = 43 N (%)	Secondary School (Gr. 2) *n* = 101 N (%)	Vocational School (Gr. 3) *n* = 139 N (%)	Grammar School (Gr. 4) *n* = 131 N (%)	University (Gr. 5) *n* = 87 N (%)	*p*-Value	Post Hoc Test
Health literacy (+) ^†^	6 (14)	35 (34)	50 (36)	54 (41)	44 (51)	<0.001	Gr.1:Gr.4 **, Gr.1:Gr.5 **, Gr.2:Gr.5 **, Gr.3:Gr.5 **, Gr.4:Gr.5 **
Compliance (+)	25 (57)	50 (49)	78 (56)	76 (58)	59 (69)	<0.001	Gr.2:Gr.5 **, Gr.3:Gr.5 **, Gr.4:Gr.5 **
Inform on psychosocial issues (+)	14 (32)	21 (21)	25 (18)	48 (37)	41 (48)	<0.001	Gr.2:Gr.4 **, Gr.2:Gr.5 **, Gr.3:Gr.4 **, Gr.3:Gr.5 **, Gr.4:Gr.5 **
Asking for clarification (+)	19 (45)	52 (51)	58 (42)	82 (63)	68 (78)	<0.001	Gr.1:Gr.4 *, Gr.1:Gr.5 **, Gr.2:Gr.3 ** Gr.2:Gr.4 **, Gr.2:Gr.5 **, Gr.3:Gr.4 ** Gr.3:Gr.5 **, Gr.4:Gr.5 **

^†^ (+), “very important”, * *p* < 0.05, ** *p* < 0.01. Abbreviations: yr, years; Gr., group.

**Table 5:** Influence of the GP’s work experience on their perception of the patient’s role.

Work Experience	None (Gr. 1) *n* = 9 N (%)	1–2 yrs. (Gr. 2) *n* = 8 N (%)	3–4 yrs. (Gr. 3) *n* = 9 N (%)	5–9 yrs. (Gr. 4) *n* = 18 N (%)	10–19 yrs. (Gr. 5) *n* = 22 N (%)	20+ yrs. (Gr. 6) *n* = 42 N (%)	*p*-Value	Post Hoc Test
Openness/honesty (+)	7 (78)	7 (87)	7 (87)	10 (56)	17 (77)	23 (55)	<0.001	Gr.3:Gr.4 *, Gr.3:Gr.6 *, Gr.4:Gr.5 *, Gr.5:Gr.6 **
Inform about self-medication (+)	7 (78)	7 (87)	8 (89)	11 (61)	18 (82)	28 (67)	0.0023 *	Gr.3:Gr.4 *, Gr.4:Gr.5 *, Gr.5:Gr.6 *
Results of other consultations (+)	8 (89)	7 (87)	9 (100)	15 (82)	16 (73)	29 (69)	0.003 **	Gr.3:Gr.5 *, Gr.3:Gr.6 *

(+), “very important”, * *p* < 0.05, ** *p* < 0.01. Abbreviations: yr, years; Gr., group.

## Data Availability

The datasets used and/or analyzed during the current study are available from the corresponding author upon reasonable request.
